# Development and control of a robotic assistant walking aid for fall risk reduction

**DOI:** 10.3389/frobt.2025.1646803

**Published:** 2025-10-15

**Authors:** Marcel Naderer, Yeongjae Kim, Tae-Hyoung Kim, Yeongmi Kim

**Affiliations:** 1 Department of Medical and Health Technologies, The Entrepreneurial School® (MCI), Innsbruck, Austria; 2 School of Mechanical Engineering, Chung-Ang University, Seoul, Republic of Korea

**Keywords:** fall prevention, robotic assistant, balance stabilization, particle swarm optimization algorithm, cascade control

## Abstract

Falls are a major risk factor among the elderly, often resulting in injuries that compromise independence and quality of life. Conventional walking aids lack active stabilization capabilities and are therefore limited in effectively preventing balance-related accidents. This paper presents the design and control of a smart robotic assistant aimed at reducing fall risk in elderly users by providing real-time balance support. The proposed system uses a wearable inertial measurement unit to detect postural imbalances in the sagittal (front-back) and frontal (side-to-side) planes. When instability is detected, the robotic arm generates compensatory forces or torques through linear or rotational actuators to help the user regain a stable posture. Using a cascaded control architecture, the outer loop is designed to maintain the user’s upright posture, while the inner loop ensures fast and accurate actuator performance. To enable effective and reliable control in the real system, actuator dynamics are characterized through an optimization-based system identification approach, resulting in transfer function models with over 98% accuracy. Based on these models, PID controllers are optimally tuned using an optimization algorithm to ensure fast and accurate corrective action. The system effectively returns the user to a stable position within 2.3 ± 0.3 s for linear actuation (with a response time of 120 ± 10 ms) and 2.2 ± 0.2 s for rotary actuation (with a response time of 140 ± 15 ms), providing safe posture return during imbalance events. To further enhance safety, an automatic braking mechanism immobilizes the walking aid during corrective maneuvers. Experimental validation demonstrates the system’s effectiveness in detecting and correcting postural imbalances in both the sagittal and frontal planes under dynamic conditions. These results highlight the potential for enhancing mobility, safety, and therapeutic support for older adults, contributing to the advancement of assistive fall-prevention technologies.

## 1 Introduction

Advances in medical technology and improvements in living standards have led to a significant increase in life expectancy, contributing to the rapid acceleration of global population aging. According to the World Health Organization, the number of individuals aged 60 years and older is projected to more than double by 2050, reaching approximately 2.1 billion worldwide (World Health Organization, 2024). This demographic shift is driving a substantial rise in the demand for healthcare and social services; however, the availability of specialized personnel, long-term care facilities, and medical infrastructure remains insufficient to meet the growing needs of the elderly population (Jones and Dolsten, 2024). In addition to the challenges associated with healthcare provision, age-related physiological changes further exacerbate the vulnerability of older adults.

Sarcopenia, characterized by the progressive loss of muscle mass and strength, alongside joint degeneration, severely impairs mobility. Furthermore, declines in vestibular and neurological function diminish balance control, increasing the risk of falls (Agrawal et al., 2020; Johnson et al., 2020). Age-related declines in physical function also significantly elevate the risk of falls among older adults. With advancing age, muscle strength diminishes, joint flexibility decreases, and balance control weakens, collectively increasing vulnerability to falls even during routine daily activities (Xing et al., 2023). Approximately one-third of individuals aged 65 years and older experience at least one fall each year (World Health Organization, 2008; Cuevas-Trisan, 2019). This prevalence highlights the pervasive nature of falls as a major public health concern in aging societies. Falls among older adults often result in severe injuries such as fractures, traumatic brain injuries, and prolonged immobility, leading to a substantial loss of independence and a marked decline in overall quality of life (Centers for Disease Control and Prevention, 2024). Beyond the immediate physical consequences, falls frequently instill a fear of falling again, causing older individuals to avoid social activities and limit their mobility, which in turn accelerates physical deterioration and psychological distress (Xing et al., 2023; Tabacchi et al., 2025). Moreover, serious falls are associated with increased mortality rates and impose significant financial burdens on both individuals and healthcare systems (Li Y. et al., 2023).

To mitigate the devastating impact of falls, various assistive devices such as canes and walking aids have been developed. They are particularly valued for their ability to enhance balance, provide greater walking stability, and reduce the load on the user’s lower extremities (Miyasike-daSilva et al., 2013; Sehgal et al., 2021). By offering mechanical support during ambulation, walking assistive devices play a critical role in helping older adults maintain their mobility, autonomy, and social engagement, ultimately contributing to fall prevention and improved quality of life. Walking aids are widely utilized mobility aids due to their simple mechanical structure, ease of operation, and ability to provide physical support during ambulation (Sutera et al., 2025). By distributing a portion of the user’s body weight and offering a stable frame for support, these devices assist individuals in maintaining upright posture and enhancing walking stability (Bateni and Maki, 2005). However, despite their widespread use and basic effectiveness, conventional walking aids have inherent limitations. While they passively support balance, they lack the ability to actively monitor a user’s stability or intervene to prevent falls (Nickerson et al., 2023). Therefore, they offer only limited protection against sudden losses of balance or unanticipated environmental disturbances. Particularly in dynamic or complex environments, where real-time adjustments are critical, passive walking aids can fail to provide adequate safety. In response to these shortcomings, there has been a growing interest in the development of active assistant walking aids, also known as smart robotic walking aids (Shin et al., 2016; Zhao et al., 2020; Sierra M. et al., 2024). Some of these advanced devices are equipped with sensors such as inertial measurement units (IMUs), capable of detecting real-time changes in the user’s posture and stability (Zhao et al., 2020; Sierra M. et al., 2024; Ferrari et al., 2024). By integrating active control mechanisms, these walking aids can effectively respond to balance disturbances, stabilize the user, and thereby significantly reduce the risk of falls. In addition, commercial smart walkers have recently become available. For example, the Camino Walker, developed by Camino Robotics, provides AI-powered gait monitoring, obstacle detection, and motorized drive assistance to support elderly users during daily tasks. Although these systems emphasize gait monitoring and assistive braking, they lack the ability to apply corrective forces to counteract balance deviations (Camino Robotics, 2025). As the aging population increases, there is a growing need for smart, adaptive mobility aids that not only provide passive support but also actively reduce fall risk.

In recent years, there has been growing interest in enhancing traditional walking aids with sensor-based technologies to improve fall detection and user safety. Several systems have been developed that utilize IMUs to monitor posture or detect falls after they occur (Sierra M. et al., 2024; Mao et al., 2017; Huang and Garcia, 2023). In addition, robotic systems combining IMUs and force sensors have been proposed to enable real-time balance monitoring and assistive feedback (Zhao et al., 2020; Sierra M. et al., 2019; Li L. et al., 2023). To effectively control such systems, various strategies have been introduced. For instance, deep neural networks have been used to analyze lower-limb gait patterns and predict user intent, enabling robotic walkers to adjust their target position and velocity accordingly (Zhao et al., 2020). Itadera et al. implemented Model Predictive Control (MPC) in a robotic walker to proactively estimate user assistance requirements and generate optimized supportive forces in real time (Itadera et al., 2019). Although such approaches have demonstrated promising results in detecting user instability and generating corrective responses via robotic arms, their real-world applicability remains limited due to issues such as large physical size, high computational requirements, and elevated cost. Mori et al. (2024) attempted to reduce system complexity and cost by estimating user posture using a compact camera and implementing PID control via a single-board computer. However, the performance of the PID controller was constrained by manually tuned gains, which limited its applicability and overall effectiveness. In response to these limitations, this study proposes the development of a compact smart robotic assistant that improves gait stability through real-time correction of postural imbalance using optimally tuned PID controllers. The proposed system addresses key limitations of previous works by integrating automatic controller tuning, compact hardware, and rapid corrective actions suitable for practical use.

In this work, we present a novel smart walking aid that integrates IMU-based posture monitoring, a dual-actuator robotic arm, and an automatic braking system to provide real-time balance assistance for elderly users. Unlike conventional passive walking aids, our system actively detects postural deviations and applies targeted corrective forces through optimized actuator control, with brakes ensuring platform stability during intervention. The key innovation lies in the integration of optimization-based system identification using Particle Swarm Optimization (PSO) to accurately model actuator dynamics and automatically tune PID controllers, enabling precise balance correction with cost-effective hardware. This comprehensive approach bridges the gap between simulation and real-world deployment, offering a practical solution for enhancing mobility safety and independence in aging populations.

## 2 Materials and methods

### 2.1 Mechanical design

**FIGURE 1:**
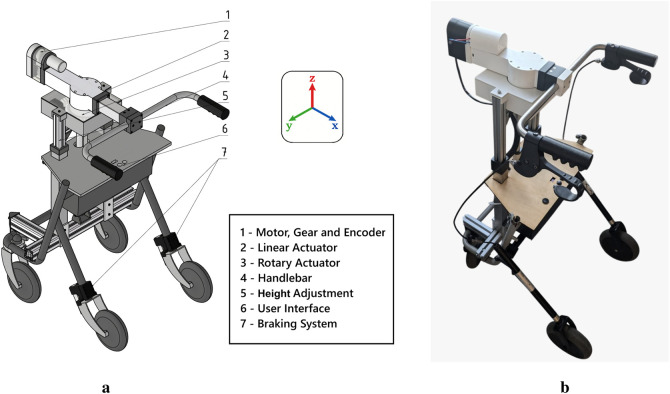
Mechanical design and final implementation of the smart robotic walking aid for supporting user balance. **(a)** CAD model illustrating the integration of linear and rotary actuators for pitch and roll compensation. **(b)** Assembled prototype of the walking aid, demonstrating the practical realization of the designed components.

The robotic arm, integrated into a conventional walking aid, is designed to compensate for user imbalance through controlled mechanical displacement. The CAD design presented in Figure 1a and, Figure 1b illustrates the assembled system. The robot features three Degrees of Freedom (DoFs): two translational (along the X and Z-axes) and one rotational (about the Z-axis). The Z-axis translation is solely used for height adjustment, while the X-axis translation and Z-axis rotation are responsible for user interaction. User imbalance is characterized by pitch (rotation around the X-axis) and roll (rotation around the Y-axis). The mechanical design prioritizes rapid dynamic response, high force output, and compact integration to ensure suitability for mobile assistive applications.

### 2.2 Balance stabilization in sagittal plane

**FIGURE 2:**
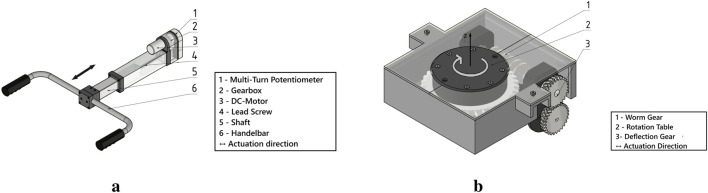
Linear and rotational actuators for compensating pitch and roll direction imbalances. **(a)** Linear actuator designed for pitch compensation. **(b)** Rotational actuator designed for roll compensations.

The customized linear actuator is responsible for compensating for pitch-direction imbalances by generating translational motion along the X-axis. A handlebar is attached to the shaft, which is shown in Figure 2a. It is driven by a brushed DC motor with an integrated high-torque gear-head to ensure sufficient force output. To achieve the required actuation speed, an additional custom-designed 1:1.5 ratio gear-head is added downstream of the motor. This combination enhances the overall drive speed without a significant reduction in torque. The rotary motion of the motor is transmitted to a high-pitch lead screw, which converts it to linear motion. A square steel shaft connected to the lead-screw nut blocks rotation and minimizes backlash. For accurate position sensing, a multi-turn potentiometer (3590P-2-203L, Bourns) is mounted adjacent to the lead screw and mechanically coupled via a 5:1 auxiliary reduction gear. This gearing allows the 10-turn potentiometer to measure the full 40-turn range of the actuator. The sensor provides accurate feedback for closed-loop control.

### 2.3 Balance stabilization in frontal plane

The rotary actuator provides roll stabilization by compensating for lateral imbalances through controlled rotation around the Z-axis and it is driven by a brushed DC motor. Due to space constraints, the motor is mounted below the worm gear, and the motion is diverted by using a 1:1 deflection gear. This allows the motor to remain integrated within the walking aid.

The rotary motion is transmitted to a worm gear that is coupled to a slewing bearing, which is located in the base of the platform. The worm gear has a high reduction ratio, which allows it to transmit high torque for the counteracting movement. An advantage of this worm gear is its self-locking behavior, which prevents back-driving, which is critical for stability during simple walking aid movements.

For the position feedback, a potentiometer is mounted coaxially on the rotation axis of the worm gear. This configuration allows accurate tracking of the rotation of the handlebar, which is necessary for the closed loop control of the rotational actuator. The design is shown in Figure 2b.

### 2.4 Electrical design

**FIGURE 3:**
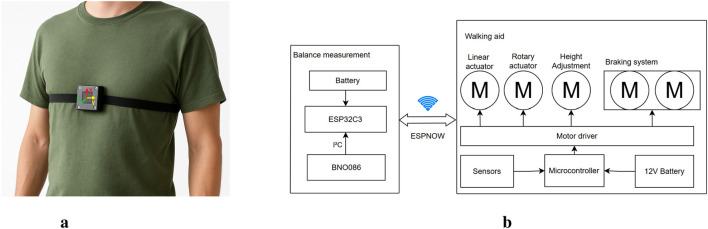
Visualization of the balance measurement system. **(a)** Placement of the IMU unit on the user’s chest. **(b)** Electrical system architecture of the smart walking aid. The balance measurement unit, consisting of a IMU, ESP32C3, and battery, communicates wirelessly via ESP-NOW with the main walking aid unit. The walking aid includes linear and rotary actuators, a braking system, motor drivers, and a micro-controller powered by a 12 V battery.

A dual micro-controller architecture is implemented based on the ESP32 NodeMCU and the Seeed ESP32C3. The ESP32 NodeMCU is responsible for processing the sensor data and controlling the motors, while the Seeed ESP32C3 manages the communication with the external balance measuring unit. Power is supplied from a 12 V rechargeable battery and regulated to different voltage levels using a buck converter to meet the requirements of the various components. Sensors include multi-turn potentiometers for position feedback, current sensors for motor monitoring, limit switches for safety and user interface buttons. All components, including micro-controllers, power regulation circuitry and sensor connections, are integrated onto a custom-designed two-layer PCB. The electrical setup is illustrated in Figure 3b.

### 2.5 Balance measurement

The user’s balance is monitored by a chest-mounted (the placement of the IMU is shown in Figure 3a) an IMU that integrates a gyroscope, accelerometer, and magnetometer.

The IMU (BNO086, Bosch) provides a resolution of 14 bits for orientation measurements and this precision corresponds to approximately 0.022° per least significant bit over a 360° range. It exceeds the required resolution of ±1°, ensuring accurate tracking of angular movements. The IMU uses an on-chip sensor fusion algorithm that combines data from the accelerometer, gyroscope, and magnetometer to compensate for drift in orientation measurements. Sensor noise and gyroscopic drift are minimized using a Kalman-based fusion approach implemented through SH-2 firmware. Before operation, an initial calibration procedure is performed. During this procedure, the user maintains a stable, upright posture while the system averages 100 consecutive measurements to define a neutral reference orientation.

The IMU continuously transmits orientation data to the walking aid’s control unit via ESP-NOW, a low-latency communication protocol based on Wi-Fi. The communication is done by a Seeed ESP32C3, where the whole setup of the measurement unit is shown in Figure 3b. This wireless setup allows real-time monitoring of pitch and roll angles without restricting the user’s mobility.

To ensure accurate measurements, the IMU goes through an initial calibration procedure prior to operation. During calibration, the user is asked to maintain a stable, upright position while the system records 100 measurements. The values are then averaged to calculate a sensor bias, which is used to detect future deviations from the neutral standing position.

The compensation thresholds are preset at ±8° for pitch and roll. If the measured orientation exceeds these limits, the system interprets this as a potential fall scenario and activates the stabilization response. The braking system is immediately engaged to immobilize the walking aid and the appropriate actuator is triggered to generate a counteracting force.

### 2.6 Braking system

**FIGURE 4:**
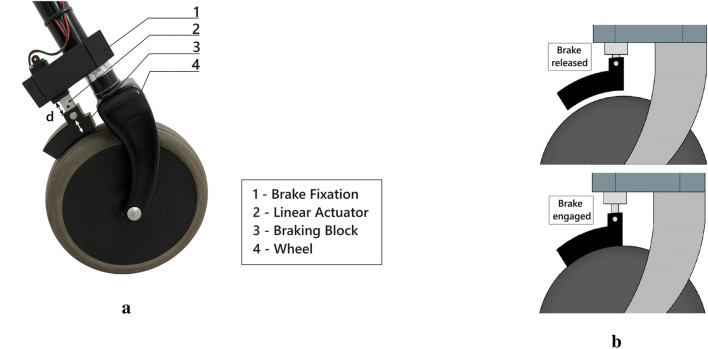
Setup of the braking system in the robotic assistant, illustrating the integration of the linear actuator for brake activation. The distance d is the actuator stroke. **(a)** Braking System. **(b)** Brake functionality.

The robotic assistant includes an electromechanical braking system. A compact linear actuator with a stroke of d = 10 mm (of which only 5 mm are required for full engagement) is used to apply mechanical force to the brake block. The setup is shown in Figure 4. When activated, the actuator pushes the brake block directly against the rear wheel, where the wheel is fully blocked. The actuator has an actuation time of 0.8 s and provides a holding force of 50 N, ensuring a quick braking response.

The braking system plays a critical role in the overall functionality of the robotic assistant, as it provides a stable base during corrective actions. Without this immobilization, any compensatory force generated by the actuators could not be transferred effectively. Therefore, the brake is automatically triggered immediately after an imbalance exceeds the defined threshold. The brake is released automatically after 5 s if no further imbalance is detected.

## 3 Control system design

### 3.1 Cascade control architecture

**FIGURE 5:**
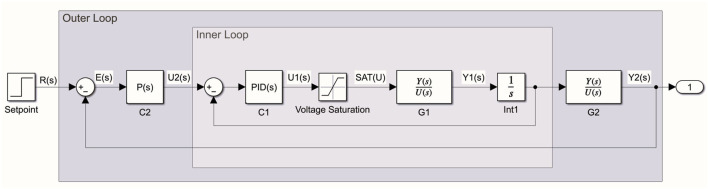
Cascade control loop of the balance stabilization system, illustrating the outer loop for user posture correction and the inner loop for precise actuator control.

A structured control architecture is developed to achieve real-time balance correction and ensure the safe and stable operation of the proposed robotic walking aid. As illustrated in Figure 5, the system utilizes a robotic arm to correct user imbalance when deviations from stable posture are detected via the IMU sensor. The control architecture adopts a two-layer cascade control scheme to effectively manage both the actuators and the user support system. The inner control loop focuses on the low-level control of the robotic arm’s actuators, which consist of a linear motor and a rotational motor. These actuators are modeled as a transfer function, denoted by $G_{1}\left(s\right)$ , representing the relationship between voltage input and velocity output. Based on this model, PID controllers are designed to perform reference position tracking for each actuator. To ensure safe operation and avoid performance degradation, voltage saturation and anti-windup mechanisms are integrated to prevent integral accumulation when the actuators are saturated. The outer control loop is responsible for determining the appropriate corrective action based on the user’s posture, as measured by the IMU. In this layer, the user is treated as a plant, denoted by $G_{2}\left(s\right)$ , and the control objective is to maintain the user’s posture within a defined stable upright position range, represented by R(s) . A proportional controller is employed in the outer loop because the posture deviation typically returns to equilibrium with fewer steady-state errors, and there is large variability in user dynamics. Integral gain was omitted as steady-state errors are minimal, and derivative gain was avoided to reduce sensitivity to high-frequency noise and unmodeled dynamics, which could induce unnecessary instability. This two-layer approach decouples actuator-level dynamics from user-level correction, allowing for both fine-grained control precision and robust response to balance disturbances.

### 3.2 System identification

To implement precise control, it is essential to accurately model the dynamic behavior of the DC motor. An accurate model plays a crucial role in representing the response characteristics of the system and allows more reliable control system design, performance evaluation, and stability analysis. In this study, the DC motor is modeled using a second-order transfer function because of its simplicity and its ability to capture the dominant dynamics of the system. Although higher-order models can provide improved accuracy, they often introduce unnecessary complexity and increase the risk of overfitting, particularly when experimental data are limited. Therefore, a general DC motor model is adopted, represented by the following transfer function: $\frac{b_{1}}{s^{2}+a_{2}s+a_{1}}$ , where the coefficients $\vec{x}={\left[a_{1},a_{2},b_{1}\right]}^{⊤}$ are identified based on experimental input-output data using an optimization-based approach. The model identification problem is formulated as an optimization problem in which the aim is to find the optimal transfer function that minimizes the error between the measured output (y) and the simulated output $\left(\hat{y}\right)$ generated by the candidate model:

$$\begin{array}{cc} & \underset{a_{1},a_{2},b_{1}}{\text{minimize}}\;J\left(\vec{x}\right):=\sqrt{\overset{n}{\underset{i=1}{\sum }}{\left(y_{i}-{\hat{y}}_{i}\right)}^{2}}\\ & \text{subject to}\;g_{j}\left(\vec{x}\right)<0,\;j=1,\ldots ,m, \end{array}$$ (1)

where n denotes the number of sampled data and m denotes the number of inequality constraints imposed in the optimization problems. To solve this optimization problem, the particle swarm optimization (PSO) algorithm is employed because of its effectiveness in handling complex objective functions and approaching the global optimum.

### 3.3 Particle swarm optimization algorithm

The PSO algorithm is a meta-heuristic optimization method inspired by the collective behavior of bird flocks and fish schools as they search for optimal positions within their groups. Each particle in the swarm represents a candidate solution and updates its position based on both its own experience and the experience of neighboring particles. The PSO algorithm employs an updating formula that models the social interactions among particles. Each particle updates its velocity and position according to the following rule:

$${\vec{v}}_{i}^{k+1}=c_{0}v_{i}^{k}+c_{1}r_{1,i}^{k}\left({\vec{x}}_{\text{pbest},i}^{k}-x_{i}^{k}\right)+c_{2}r_{2,i}^{k}\left({\vec{x}}_{\text{gbest}}^{k}-x_{i}^{k}\right),$$

$${\vec{x}}_{i}^{k+1}=x_{i}^{k}+v_{i}^{k+1},$$

where i denotes the index of the particle, and k represents the iteration step. The vector ${\vec{v}}_{i}^{k}$ is the velocity of particle i at iteration k , and ${\vec{x}}_{i}^{k}$ is its position. The vector ${\vec{x}}_{\text{pbest},i}^{k}$ represents the best position found by particle i up to the current iteration k , while ${\vec{x}}_{\text{gbest}}^{k}$ denotes the best position found by the entire swarm up to the iteration k . The parameters $c_{0}$ , $c_{1}$ , and $c_{2}$ are hyper-parameters that determine the influence of inertia, the particle’s personal best position, and the global best position identified by the swarm, respectively. The random variables $r_{1,i}^{k}$ and $r_{2,i}^{k}$ are uniformly distributed in the range [0,1], introducing stochastic effects into the update process. When the design variables of an optimization problem are defined as the position vectors of individual particles, the PSO updating rule enables these variables to be iteratively updated toward better solutions. Through this mechanism, the PSO algorithm can efficiently guide the optimization process toward the global optimum.

In this study, the actuator model is identified using the PSO algorithm. The optimization problem for model identification is formulated as described in Equation 1, where the design variables to be estimated are $\left\{a_{1},a_{2},b_{1}\right\}$ . These design variables are assigned as the position vectors of the particles in the PSO algorithm. According to the PSO updating rule, each particle iteratively adjusts its position vector to minimize the objective function, defined as $\sqrt{\sum_{i=1}^{n}{\left(y_{i}-{\hat{y}}_{i}\right)}^{2}}$ , which is the discrepancy between the measured output and the output simulated by the candidate model. To enhance the exploration capability and prevent premature convergence to local minima, the distributed particle swarm optimization (DPSO) algorithm with a cyclic network topology (Kim et al., 2022) is employed. The detailed procedure for model identification using the DPSO algorithm is described as follows:

1. Initialization: The following initial parameters are set: the number of particles used for exploration $N_{p}$ ; the number of neighbors with which each particle exchanges information $N_{s}$ ; the hyperparameters for updating the design variables $\left\{c_{0},c_{1},c_{2}\right\}$ ; the maximum number of iterations, maxiter ; and the search range for each design variable, defined by the inequality constraints $p_{\min,\ell }\leq p_{\ell }\leq p_{\max,\ell }$ for $\ell =1,2,\ldots ,N_{d}$ , where $\vec{p}\in R^{N_{d}}$ and $N_{d}=3$ for the model identification problem (1). These boundaries can be expressed as a set of inequality functions $g_{j}\left(\vec{p}\right)<0$ (e.g., $g_{1}=p_{\min,1}-p_{i,1}<0$ for ${\vec{p}}_{i}={\left[p_{i,1},p_{i,2},p_{i,3}\right]}^{⊤}$ ). The initial position and velocity vectors of each particle i are randomly generated within the predefined search space as follows:

$${\vec{p}}_{i}^{0}={\vec{p}}_{\min}+R_{0,i}\left({\vec{p}}_{\max}-{\vec{p}}_{\min}\right),$$

$${\vec{v}}_{i}^{0}=\vec{0},$$

where $R_{0,i}\in R^{N_{d}\times N_{d}}$ is a diagonal matrix whose elements are uniformly distributed in the range [0,1], and $\vec{0}\in R^{N_{d}}$ denotes the zero vector.

2. Evaluation of the fitness value: Each particle’s position vector ${\vec{p}}_{i}$ is used to determine the corresponding design parameters (i.e., ${\left[p_{1},p_{2},p_{3}\right]}^{⊤}\to {\left[a_{1},a_{2},b_{1}\right]}^{⊤}$ ), which define the coefficients of the transfer function. Using the transfer function and the given input data, the simulated system output $\vec{\hat{y}}$ is obtained. The fitness value of each particle is then evaluated based on the objective function. To handle boundary constraints, the fitness value is recalculated as:

$$L\left({\vec{p}}_{i}\right):=\left\{\begin{array}{cc} g_{\max}\left({\vec{p}}_{i}\right)\; & \text{if}\;g_{\max}\left({\vec{p}}_{i}\right)\geq 0,\\ f_{v}\left({\vec{p}}_{i}\right)\; & \text{otherwise}, \end{array}\right.$$

where $g_{\max}\left({\vec{p}}_{i}\right):=\max\left[g_{1}\left({\vec{p}}_{i}\right),g_{2}\left({\vec{p}}_{i}\right),\ldots ,g_{m}\left({\vec{p}}_{i}\right)\right]$ , and $f_{v}\left({\vec{p}}_{i}\right):=\arctan\left\{J\left({\vec{p}}_{i}\right)\right\}-\frac{\pi }{2}$ . If the particle position vector ${\vec{p}}_{i}$ violates any of the predefined constraints (i.e., it lies outside the search boundaries), then at least one constraint function becomes non-negative, resulting in $g_{\max}\left({\vec{p}}_{i}\right)\geq 0$ . In this case, the particle is assigned a positive fitness value, indicating that it resides in the infeasible region. In contrast, if the particle remains within the feasible region, all constraint functions yield strictly negative values, and the fitness value is calculated using the transformed function $f_{v}\left({\vec{p}}_{i}\right)$ , which always yields a negative value. Through this mechanism, the feasible and infeasible regions are distinctly separated into the negative and positive domains of the fitness value space, respectively. As a result, the optimization algorithm—designed to minimize the fitness value—naturally guides the particles toward the feasible search region.

3. Evaluation of optimal positions: The individual best position of each particle and the social best position ${\vec{p}}_{\text{sbest},i}^{k}$ —defined as the best position among neighbors of the j -th particle (ranging from $j-\frac{N_{s}}{2}$ to $j+\frac{N_{s}}{2}$ , where $N_{s}$ denotes the neighborhood size of the distributed cyclic neighborhood topology)—are both determined based on fitness values during the k -th iteration. These best positions are determined using the following mathematical expressions:

$${\vec{p}}_{\text{pbest},i}^{k}\leftarrow \arg\underset{\vec{p}\in \left\{{\vec{p}}_{i}^{m}|m=1,2,\ldots ,k\right\}}{\min}L\left(\vec{p}\right),$$

$${\vec{p}}_{\text{sbest},i}^{k}\leftarrow \arg\underset{\vec{p}\in \left\{{\vec{p}}_{m}^{k}|m=i-\frac{N_{s}}{2},\ldots ,i+\frac{N_{s}}{2}\right\}}{\min}L\left(\vec{p}\right),$$

where ${\vec{p}}_{i}^{k}:={\vec{p}}_{\left(i-1\;\text{mod}\;N_{p}\right)+1}^{k}$ for i<1 or $N_{p}+1\leq i$ to implement cyclic indexing.

4. Update of position and velocity vectors: The velocity and position vectors of each particle are updated according to the DPSO update rules as follows:

$${\vec{v}}_{i}^{k+1}=c_{0}{\vec{v}}_{i}^{k}+c_{1}r_{1,i}^{k}\left({\vec{p}}_{\text{pbest},i}^{k}-{\vec{p}}_{i}^{k}\right)+c_{2}r_{2,i}^{k}\left({\vec{p}}_{\text{sbest},i}^{k}-{\vec{p}}_{i}^{k}\right),$$

$${\vec{p}}_{i}^{k+1}={\vec{p}}_{i}^{k}+{\vec{v}}_{i}^{k+1},$$

where $r_{1,i}^{k}$ and $r_{2,i}^{k}$ are random scalar values distributed uniformly in [0,1].

5. Termination criterion: If the termination criterion is satisfied (e.g., k=maxiter ), the optimal solution ${\vec{p}}^{*}$ is obtained, which is defined as:

$${\vec{p}}^{*}\leftarrow \arg\underset{\vec{p}\in \left\{{\vec{p}}_{i}^{k}|i=1,2,\ldots ,N_{p};\;k=1,2,\ldots ,maxiter\right\}}{\min}L\left(\vec{p}\right).$$

Otherwise, return to Step 2.

**FIGURE 6:**
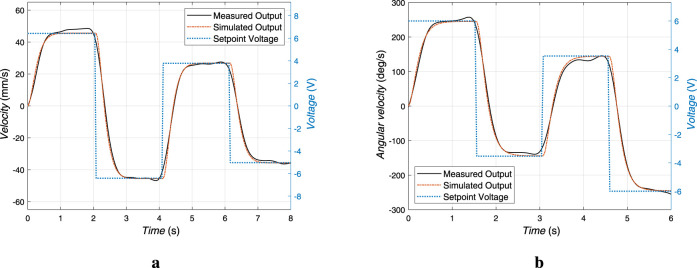
Validation of actuator models through comparison of measured and simulated velocity responses. The RMSE between the measured with a low pass filter and simulated responses is **(a)** 2.17 mm/s for the linear actuator and **(b)** 8.798 deg/s for the rotary actuator.

Based on the procedure described above, the model identification is carried out using the DPSO algorithm. The parameter values used in the DPSO algorithm are summarized as follows: $N_{p}=200$ ; $N_{s}=20$ ; $\left\{c_{0},c_{1},c_{2}\right\}=\left\{0.7298,1.4962,1.4962\right\}$ ; maxiter=1000 ; ${\vec{p}}_{\min}={\left[-1000,-1000,-1000\right]}^{⊤}\in R^{3}$ ; and ${\vec{p}}_{\max}={\left[1000,1000,1000\right]}^{⊤}\in R^{3}$ . To identify the actual actuator models, experimental input-output data are collected from the real system. Specifically, the blue dotted lines in Figure 6 represent the setpoint voltage input, while the black solid lines depict the measured velocity output obtained from the actuator sensors. As a result of the model identification, the identified transfer functions for the linear actuator and rotary actuator are respectively derived as.

$$G{\left(s\right)}_{\text{Linear}}=\frac{332.0120}{s^{2}+12.4316s+43.1780},$$

$$G{\left(s\right)}_{\text{Rotary}}=\frac{2651.4155}{s^{2}+16.2155s+64.9365}.$$

To validate the fidelity of the identified models, the simulated outputs generated from these transfer functions are compared against the measured data under the same input conditions. The orange dash-dotted lines in Figures 6a,b represent the simulated outputs for the linear and rotary actuators, respectively. To quantitatively assess the accuracy of the model identification, the coefficient of determination is calculated (Devore, 2013). This statistical metric indicates how well the simulated outputs replicate the actual system behavior. The computed values for the linear and rotational models are 98.70% and 99.19% , respectively. These high values confirm that the identified models accurately capture the dynamic response of the actuators and are sufficiently precise for subsequent controller design and stability analysis. The fidelity of these models ensures that the resulting control system can perform reliably under real-time operating conditions.

### 3.4 PID controller tuning using the DPSO algorithm

In this study, a proportional-integral-derivative (PID) controller-based feedback strategy is employed to improve the tracking performance of the robotic arm actuator. Given the critical role of accurate tracking in maintaining user postural stability, the PID gain values are optimally tuned using a metaheuristic optimization approach. The tuning process is guided by a performance-based objective function that considers three key metrics: overshoot (OS) , settling time $\left(T_{s}\right)$ , and the integral of time-weighted absolute error (ITAE). Overshoot and settling time are included to ensure minimal deviation and fast convergence, while the ITAE term penalizes long-lasting steady-state errors. The objective function is formulated as follows: $J=OS+T_{s}+\int_{0}^{T}t|e\left(t\right)|\;dt$ , where e(t) is the tracking error and T is the total evaluation time. The objective value is computed by simulating the closed-loop step response of the actuator model under a candidate PID gain set ${\vec{p}}_{i}\to {\left[K_{p},K_{i},K_{d}\right]}^{⊤}$ , where each gain set corresponds to a position vector of a particle in the DPSO algorithm. To ensure feasible solutions that guarantee system stability and control performance, several constraints are imposed during the optimization: i ${\vec{p}}_{\min}\leq {\vec{p}}_{i}\leq {\vec{p}}_{\max}$ to bound the design parameter ranges, ii $\max\left(R\left(\lambda_{i}\right)\right)<0$ , where $\lambda_{i}$ denotes a pole of the closed-loop system to ensure closed-loop stability, and iii OS≤10% to limit the overshoot. A key challenge in such constrained optimization problems is maintaining the balance between exploration and exploitation while strictly enforcing feasibility. To address this, a fitness evaluation method is designed to improve convergence efficiency by classifying solutions into three regions based on feasibility: infeasible due to boundary or stability violation, infeasible due to overshoot violation, and fully feasible. The fitness value $L\left({\vec{p}}_{i}\right)$ of each particle is then computed using the following formulation:

$$L\left({\vec{p}}_{i}\right):=\left\{\begin{array}{cc} \arctan\left\{g_{\max}\left({\vec{p}}_{i}\right)\right\}+\frac{\pi }{2}\; & \text{if}\;g_{\max}\left({\vec{p}}_{i}\right)\geq 0,\\ \arctan\left\{h\left({\vec{p}}_{i}\right)\right\}\; & \text{if}\;g_{\max}\left({\vec{p}}_{i}\right)<0\;\text{and}\;h\left({\vec{p}}_{i}\right)\geq 0,\\ \arctan\left\{J\left({\vec{p}}_{i}\right)\right\}-\frac{\pi }{2}\; & \text{otherwise}, \end{array}\right.$$

where $g_{\max}\left({\vec{p}}_{i}\right)$ is the maximum violation among boundary and stability constraints, $h\left({\vec{p}}_{i}\right)$ is the overshoot constraint violation (OS−10) , and $J\left({\vec{p}}_{i}\right)$ is the original objective function value for feasible solutions. This fitness calculation mechanism ensures that infeasible solutions due to boundary and stability violations yield values in the range $\left(\frac{\pi }{2},\pi \right)$ , overshoot-only infeasible solutions fall in $\left(0,\frac{\pi }{2}\right)$ , and fully feasible solutions result in values in $\left(-\frac{\pi }{2},0\right)$ . Such a mapping effectively guides the optimization algorithm toward feasible regions while allowing subtle penalization of different types of violations. The tuning process proceeds as described in Section 3.3, where the DPSO algorithm searches the gain space using this fitness function to yield optimal PID controllers that meet all design objectives and constraints. The number of design variables in the optimization problem is three, corresponding to the proportional $\left(K_{p}\right)$ , integral $\left(K_{i}\right)$ , and derivative $\left(K_{d}\right)$ gains of the PID controller. The parameter search space for tuning is defined as ${\vec{p}}_{\min}={\left[0,0,0\right]}^{⊤}\in R^{3}$ and ${\vec{p}}_{\max}={\left[1000,1000,1000\right]}^{⊤}\in R^{3}$ . Other optimization parameters—such as $N_{p}$ , $N_{s}$ , $c_{0},c_{1},c_{2}$ , and maxiter —are configured identically to those used in the actuator model identification process. As a result of the optimization, the optimal PID gain sets for the linear and rotary actuator models are obtained as follows:

$${\left[K_{p},K_{i},K_{d}\right]}_{\text{Linear}}=\left[0.7529,\;0,\;0.1882\right],$$

$${\left[K_{p},K_{i},K_{d}\right]}_{\text{Rotary}}=\left[0.1714,\;0,\;0.0368\right].$$

For both controllers, the derivative filter time constant is set to 0.01 to reduce high-frequency noise amplification.

### 3.5 PID controller fine tuning

The performance of the smart robotic walking aid relies on the tuning of its control system to ensure fast and stable balance correction. The controller design follows a cascaded structure, where the inner loop handles fast actuator dynamics, and the outer loop maintains overall balance stability.

#### 3.5.1 Inner loop

To optimize the system’s response, the control parameters were fine-tuned to prioritize fast stabilization. The proportional (P) gain from the simulation already provided the desired balance between responsiveness and stability, both in the simulated and real-world environments.

In the simulation, the integral (I) component was set close to zero because the simulated environment did not exhibit steady-state errors. The lack of friction in the virtual model allowed for precise control without the need for integral correction. During real-world tests, friction in the 3D-printed gear mechanism introduced a permanent steady-state error. To compensate for this effect, an I component was added to the controller in the system.

The derivative (D) values required a small adjustment when transitioning from simulation to real-world application. This change was primarily due to unmodeled dynamic effects such as mechanical friction, vibration, and sensor noise that were not present in the idealized simulation model. The following PID parameters were implemented in the system to ensure optimal balance control:

$${\left[K_{p},K_{i},K_{d}\right]}_{\text{Linear}}=\left[0.7529,\;0.396,\;0.265\right],$$

$${\left[K_{p},K_{i},K_{d}\right]}_{\text{Rotary}}=\left[0.1736,\;0.1121,\;0.0508\right]$$

#### 3.5.2 Outer loop

The outer loop control is designed as a proportional-only controller. Due to the setpoint range in which the human remains in a stable state, the steady-state error has a negligible effect. The plant, represented by the user, shows significant variability. To keep the system simple and avoid unnecessary instability, the derivative term has been removed. The tuning process involves testing different values on the assembled walking aid.

### 3.6 Operational algorithm

**FIGURE 7:**
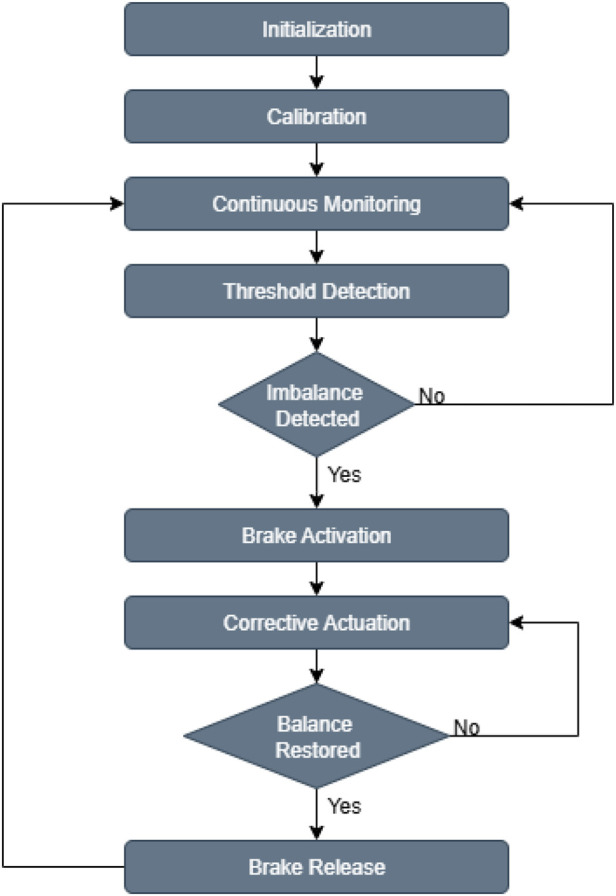
The operational algorithm of the robotic assistant walking aid for balance support.

The system begins with initialization and calibration. Next, it enters a phase of continuous monitoring, during which IMU data is collected in real time. When the system detects an imbalance by identifying that the user’s pitch or roll angle exceeds the defined limits, it immediately activates the brakes to stabilize the walker. The system then activates the linear or rotary actuator to apply a counteracting force or torque to correct the imbalance. The control loop continues until balance is restored. Once a stable posture has been reestablished and maintained for 5 s, the system releases the brakes and resumes continuous monitoring. The Algorithm is visualized in the Figure 7.

## 4 Experimental validation

A series of controlled experiments were conducted in a laboratory environment to validate the effectiveness of the proposed smart robotic assistant for balance assistance. The setup included a fully assembled robotic walking aid equipped with linear and rotary actuators, as well as a braking system. The wearable IMU was mounted on the user’s chest to provide continuous orientation feedback in the sagittal (pitch) and frontal (roll) planes. The sensor data were transmitted to the main micro-controller of the walking aid wirelessly. During testing, the postural deviations induced by the user were used to trigger the stabilization system.

The first author conducted controlled experiments in which imbalances were induced by leaning forward and to the side, exceeding the predefined thresholds of ±8° in pitch and roll. Each test run comprised three phases: 1 initiating the imbalance, 2 automatically detecting the deviation via IMU data, and 3 executing corrective actions with the actuator and braking mechanism. To objectively assess the system’s performance prior to user studies, an initial feasibility check was conducted by the first author, a healthy adult male. The experimental trials were designed to evaluate the system’s response to balance disturbances in four directions: forward, backward, and sideward. Each condition was tested six times, for a total of 24 test runs. Before each trial, the system was reset, and the participant returned to a standardized neutral standing posture to ensure consistency. The participant simulated each imbalance direction intentionally and in a controlled manner. This preliminary evaluation was intended to verify the technical feasibility of the system and to inform future user studies involving a more diverse participant group. The system recorded the following during each trial: IMU orientation, actuator positions, control input signals, and brake engagement status. System performance was evaluated using the signals and metrics, including response time, settling time, overshoot, balance recovery time, and brake activation time.

## 5 Results

### 5.1 Stability and robustness of the corrective actuation

To assess the stability and reliability of the actuation system, a series of varying setpoints were applied to the linear and rotational actuators. The system’s responses in terms of position tracking were then observed and evaluated. These tests simulated abrupt directional changes, challenging the controller’s ability to reject disturbances and maintain convergence to the target state.

**FIGURE 8:**
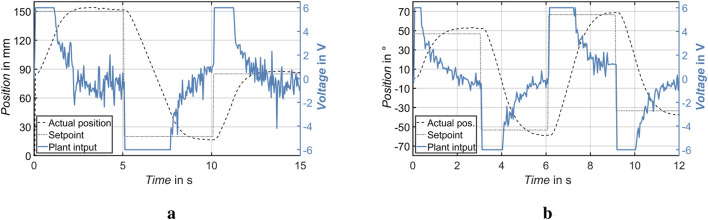
Linear and rotary actuator performance with dynamic voltage commands. Robust position tracking is maintained across multiple transitions. **(a)** Linear Actuator. **(b)** Rotary Actuator.

Figures 8a,b illustrate the system behavior for the rotational and linear actuators. In both cases, the actual position closely follows the commanded setpoint. The system remained stable throughout the tests and recovered quickly from transients, demonstrating its robustness against input variation and mechanical backlash. The defined set point ranges shown in Figures 8a,b are much greater than the actual usage range of the smart walking aid. Therefore, the device operates at maximum velocity when the desired set point changes significantly.

### 5.2 Actuator performance

The performance of both the linear and rotary actuators was evaluated with respect to response speed, positional accuracy, and stability. These parameters are critical for ensuring compensation during one imbalance. For these tests, the brakes are fully engaged to focus only on actuator performance.

#### 5.2.1 Linear actuator

**FIGURE 9:**
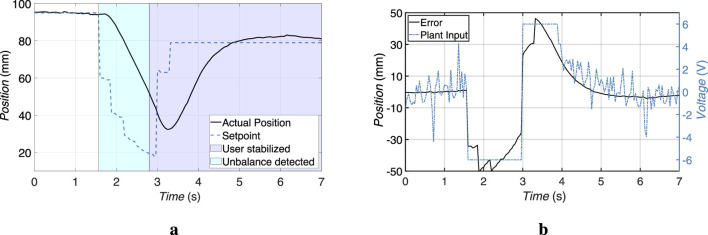
Experimental results of the linear actuator response during tilt compensation following the detection of user imbalance. **(a)** Comparison between the setpoint and the actual actuator position. **(b)** Corresponding position tracking error input and plant input applied to the actuator.

The linear actuator responsible for tilt compensation was tested while an imbalance was detected. The user leaned back and the actuator response was recorded. The actuator responded quickly, with a settling time of less than 1.4 s for the person to stabilize. The velocity measured in this test was 22.2 mm/s. The built-in high pitch lead screw, combined with a high gear ratio, allowed for rapid conversion of motor torque into linear motion while maintaining accuracy. The system is aggressively tuned to prioritize rapid stabilization, resulting in a slight overshoot during corrective actions. This adjustment ensures that the system will return the user to a stable position as quickly as possible. The result is shown in the Figure 9. The overshoot observed during the corrective action is considered acceptable because the primary goal of the system is to stop the fall immediately, not to achieve a perfectly balanced position. In addition, the overshoot does not create an imbalance in the other direction because the user is already tilting backwards.

#### 5.2.2 Rotary actuator

**FIGURE 10:**
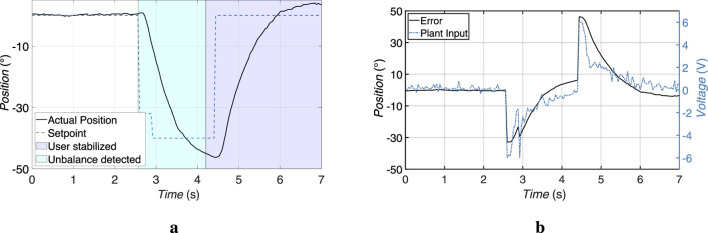
Experimental results of the rotary actuator response during tilt compensation following the detection of user imbalance. **(a)** Comparison between the setpoint and the actual actuator position. **(b)** Corresponding position tracking error and control input applied to the actuator.

The rotary actuator, which is responsible for compensating for imbalances in the roll direction, was evaluated under various lateral disturbance scenarios. The actuator’s task is to rotate the handlebar assembly about the Z-axis to counteract unwanted tilting by the user. To validate its performance a fall to the right was simulated and the actuator’s response was recorded. The actuator features a brushed DC motor coupled to the worm gear that provides both torque amplification and a self-locking effect, ensuring mechanical stability even when the motor is not powered. The response time to the required position was 1.8 s and the measured speed was 66.6 °/s . The result is displayed in the Figure 10. The overshoot observed during the rotary actuator’s corrective action is also considered acceptable because the primary goal of the system is to quickly counteract the lateral imbalance rather than to achieve an accurate balance control.

### 5.3 Balance recovery evaluation

To evaluate the real-time stabilization capabilities of the system, controlled tests were performed to simulate balance disturbances. Two primary types of disturbances were presented: pitch disturbances, representing forward or backward leaning, and roll disturbances, simulating side falls.

#### 5.3.1 Pitch disturbances

In pitch disturbance scenarios, the user leaned forward to exceed the predefined pitch threshold of ±8°. This was detected by the chest-mounted IMU. The system responded by activating the linear actuator, which generated a corrective force along the X-axis to restore the user’s upright posture.

**FIGURE 11:**
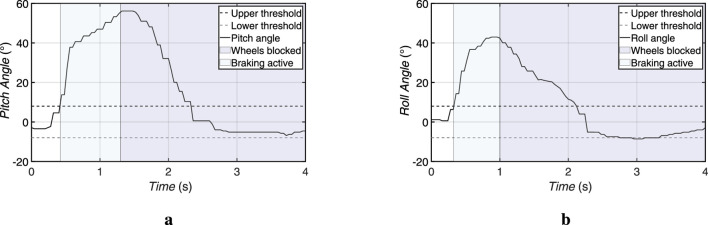
Experimental results of the actuator responses during tilt compensation after detecting user imbalance. **(a)** Linear actuator response to a forward imbalance, displaying the pitch angle and braking behavior. **(b)** Rotary actuator response to a lateral imbalance, showing the roll angle and brake activation.

At the same time, the electromechanical brake was activated to lock the wheels of the walking aid. The brake activation occurred within approximately 5 s and then automatically released if no further unbalance was detected. The linear actuator reached its target position in less than 2.3 s (with a response time of 120 ms), returning the user to a stable position. The measured results are illustrated in Figure 11a.

#### 5.3.2 Roll disturbances

For the roll recovery tests, the user was instructed to shift the weight laterally to one side, exceeding the ±8.0° roll threshold. The system responded by activating the rotary actuator, which rotated the handlebar assembly around the Z-axis to apply a counteracting torque.

As with the pitch response, the braking system was immediately activated to prevent the walking aid from moving during the correction. The activation time of the brakes equals 0.7 s. The rotary actuator completed its stabilizing motion in approximately 2.2 s (with a response time of 140 ms), ensuring that lateral deviation was quickly compensated. The self-locking characteristic of the worm gear maintained the corrected position without any additional power from the motor. The results can be seen in the Figure 11b.

## 6 Discussion

This work presents a robotic assistive walking device that integrates a compact IMU sensor and cascade control system into a walker-sized platform, enabling real-time imbalance detection and correction. The system aims to reduce the risk of falls in older adults while maintaining the size and ease of use of a conventional walker - making it practical for everyday use without the physical or cognitive strain often associated with high-end robotic devices.

A study on sensor-based walkers (Zhao et al., 2020) shows that the developed walker works well to protect users from external disturbances by using mechanical designs and advanced sensors such as IMU, LIDAR, and infrared cameras. This system shows success in assisting gait, assisting posture recovery from sitting or falling, and activating braking mechanisms to prevent further injury. However, such a system tends to be bulky, expensive, and reactive - intervening after a fall has begun rather than preventing it through early balance correction. These limitations hinder their widespread use, especially among the elderly who require simple and lightweight solutions.

In contrast, the proposed system offers a compact and familiar mechanical configuration similar to standard walkers, promoting ease of use for elderly users. The unique advantage of the proposed walker over existing models on the market is its combination of compactness and stabilization through the use of counteracting forces. While most commercially available walkers are either bulky to handle counteracting forces (Li L. et al., 2023) or remain slim but lack active balance assistance capabilities (Zhao et al., 2020; Sierra M. et al., 2019), this design achieves both. The IMU sensor is lightweight and easy to wear, and provides continuous balance monitoring without the need for complex calibration. This system also integrates optimization-based system identification and control. The Particle Swarm Optimization algorithm uses experimental data to identify the robot arm’s actuator models and optimally tune the PID gains. As a result, the actuators apply compensating forces quickly and accurately, achieving safe reference tracking with no vibration even under input saturation. These results take into account the low-cost hardware used, highlighting the system’s potential for low-cost deployment. The self-locking worm gear and automatic brake enhance the system’s ability to stabilize the user during corrective actions. These features enable the base to remain stationary while the robotic arm assists the user in regaining their balance. However, the use of a chest-mounted IMU sensor introduces variability due to differences in user height and posture, resulting in inconsistent tilt angle measurements. Consequently, identical physical postures may produce different sensor outputs for different users, which could trigger premature or delayed corrective responses. Additionally, the braking system is currently only implemented on the back wheels. In forward fall scenarios, where most of the user’s weight shifts forwards, the lack of front-wheel braking reduces the system’s ability to fully immobilize the walker. The reliance on 3D-printed components introduces play and insufficient stiffness under dynamic loads, which may compromise control accuracy and durability in real-world use. By incorporating real-time sensing, optimized control, and automated braking into a compact and intuitive device, the proposed system provides a proactive approach to fall prevention while maintaining user comfort and independence. Future research should focus on implementing machine learning algorithms that could significantly improve the accuracy of sensor detection, allowing for more reliable fall prediction. A more advanced braking system, ideally including braking on all wheels rather than just the back wheels, would improve stability, particularly in forward fall scenarios. Conducting user studies with elderly individuals would provide valuable insights into the effectiveness of the device in real-world settings. In addition, replacing 3D-printed components with more robust and durable materials could reduce backlash and improve the structural integrity of the system, increasing both stability and longevity. These improvements could make the system a practical, affordable, and intelligent mobility aid. This paper presents the design and experimental validation of a robotic assistant for real-time fall prevention for elderly users. The system combines sensor-based position monitoring, a cascade control architecture, and electromechanical braking to detect and correct pitch and roll imbalances. Experimental results confirmed the system’s ability to respond quickly to instability scenarios.

Future work will focus on improving sensor adaptability by addressing variations in IMU placement due to differences in user height. To achieve more stable and consistent position measurements, machine learning techniques will be used to compensate for different user heights and provide more accurate measurements. The braking system will be improved to provide a stronger response during forward falls. In addition, prototype components will be replaced with manufactured parts to reduce mechanical backlash and improve overall system stability. Future developments include the integration of adaptive thresholds and extended testing with target user groups in real-world environments to evaluate missed improvements.

## Data Availability

The raw data supporting the conclusions of this article will be made available by the authors, without undue reservation.
